# Influence of a novel, versatile bifunctional chelator on theranostic properties of a minigastrin analogue

**DOI:** 10.1186/s13550-015-0154-7

**Published:** 2015-12-15

**Authors:** Joachim Pfister, Dominik Summer, Christine Rangger, Milos Petrik, Elisabeth von Guggenberg, Paolo Minazzi, Giovanni B. Giovenzana, Luigi Aloj, Clemens Decristoforo

**Affiliations:** Department of Nuclear Medicine, Innsbruck Medical University, Anichstrasse 35, A-6020 Innsbruck, Austria; Institute of Molecular and Translational Medicine, Faculty of Medicine and Dentistry, Palacky University Olomouc, Olomouc, Czech Republic; CAGE Chemicals srl, Novara, Italy; DSF, Università del Piemonte Orientale “A. Avogadro”, Novara, Italy; Division of Nuclear Medicine, Istituto Nazionale Tumori, “Fondazione G. Pascale”-IRCCS, Napoli, Italy

## Abstract

**Background:**

6-[Bis(carboxymethyl)amino]-1,4-bis(carboxymethyl)-6-methyl-1,4-diazepane (AAZTA ) is a promising chelator with potential advantages over 1,4,7,10-tetraazacyclododecane-1,4,7,10-tetraacetic acid (DOTA) for radiopharmaceutical applications. Its mesocyclic structure enables fast radiolabelling under mild conditions with trivalent metals including not only ^68^Ga for positron emission tomography (PET) but also ^177^Lu and ^111^In for single-photon emission computed tomography (SPECT) and radionuclide therapy. Here, we describe the evaluation of a bifunctional AAZTA derivative conjugated to a model minigastrin derivative as a potential theranostic agent.

**Methods:**

An AAZTA derivative with an aliphatic C_9_ chain as linker was coupled to a minigastrin, namely [AAZTA^0^, D-Glu^1^, desGlu^2–6^]-minigastrin (AAZTA-MG), and labelled with ^68^Ga, ^177^Lu and ^111^In. The characterisation in vitro included stability studies in different media and determination of logD (octanol/PBS). Affinity determination (IC_50_) and cell uptake studies were performed in A431-CCK2R cells expressing the human CCK2 receptor. μPET/CT and ex vivo biodistribution studies were performed in CCK2 tumour xenograft-bearing nude mice and normal mice.

**Results:**

AAZTA-MG showed high radiochemical yields for ^68^Ga (>95 %), ^177^Lu (>98 %) and ^111^In (>98 %). The logD value of −3.7 for both [^68^Ga]- and [^177^Lu]-AAZTA-MG indicates a highly hydrophilic character. Stability tests showed overall high stability in solution with some degradation in human plasma for [^68^Ga]- and transchelation towards DTPA for and [^177^Lu]-AAZTA-MG. An IC_50_ value of 10.0 nM was determined, which indicates a high affinity for the CCK2 receptor. Specific cell uptake after 60 min was >7.5 % for [^68^Ga]-AAZTA-MG and >9.5 % for [^177^Lu]-AAZTA-MG, comparable to other DOTA-MG-analogues. μPET/CT studies in CCK2 receptor tumour xenografted mice not only revealed high selective accumulation in A431-CCK2R positive tumours of ^68^Ga-labelled AAZTA-MG (1.5 % ID/g in 1 h post injection) but also higher blood levels as corresponding DOTA-analogues. The ^111^In-labelled peptide had a tumour uptake of 1.7 % ID/g. Biodistribution in normal mice with the [^177^Lu]-AAZTA-MG showed a considerable uptake in intestine (7.3 % ID/g) and liver (1.5 % ID/g).

**Conclusion:**

Overall, AAZTA showed interesting properties as bifunctional chelator for peptides providing mild radiolabelling conditions for both ^68^Ga and trivalent metals having advantages over the currently used chelator DOTA. Studies are ongoing to further investigate in vivo targeting properties and stability issues and the influence of spacer length on biodistribution of AAZTA.

**Electronic supplementary material:**

The online version of this article (doi:10.1186/s13550-015-0154-7) contains supplementary material, which is available to authorized users.

## Background

With the increasing number of applications for peptide-based radiopharmaceuticals, new radiolabelling strategies are of high interest in particular for “theranostic” agents [1], which combine the modalities of therapy and diagnostic imaging [2]. The theranostic principle is unique for radiopharmeuticals by exchanging, for example ^68^Ga for diagnostic purposes by ^177^Lu as a therapeutic counterpart for the same targeting vector, such as a peptide, thereby translating the diagnostic molecular characterisation into a treatment opportunity for the patient. One challenge in this case is varying labelling properties for different radionuclides. Current chelators like 1,4,7,10-tetraazacyclododecane-1,4,7,10-tetraacetic acid (DOTA) have certain limitations [3], in particular providing different coordinations for different metals and requiring high temperatures for radiolabelling. Other chelators such as 1,4,7-triazacyclononane, 1-glutaric acid-4,7 acetic acid (NODAGA) [4] have shown the potential for convenient preparation of ^68^Ga-radiopharmaceuticals at room temperature; however, they cannot be used for other trivalent metals, in particular, those for radionuclide therapy applications. A novel and versatile alternative bifunctional chelator for radiopharmaceutical applications is 6-[bis(carboxymethyl)]amino]-6-(9-carboxynonyl)-1,4-diazepane-1,4-diacetic acid, tetra-*t*-butyl ester (AAZTA) [5]. AAZTA is a heptadentate ligand, based on the perhydro-1,4-diazepine scaffold enabling rapid metal coordination under mild conditions. It was first developed for magnetic resonance imaging (MRI) applications as a chelator for gadolinium [6], but showed significant affinity for several other metal ions [7]. Recently AAZTA has been proposed as potential chelator for ^68^Ga and PET applications [8] and has been used for the development of an RGD peptide conjugate as a combined PET/MRI probe radiolabelled with ^68^Ga [9].

Advantages of the AAZTA chelator are based on its mesocyclic structure which enables labelling of different radioisotopes at room temperature (RT). This allows application also for heat or oxidation sensitive peptides or other biomolecules.

We were not only interested in the application of AAZTA as bifunctional chelator for potential theranostic applications, allowing radiolabelling with ^68^Ga for positron emission tomography (PET), but also other trivalent radiometals such as ^111^In for single-photon emission computed tomography (SPECT) and ^177^Lu for therapeutic applications. We chose the minigastrin11 (MG11) analogue ([D-Glu^1^, desGlu^2–6^]^−^minigastrin, minigastrin = Glu-Glu-Glu-Glu-Glu-Glu-Ala-Tyr-Gly-Trp-Met-Asp-Phe-NH_2_) as a model targeting sequence which has been widely characterised with other chelators including HYNIC and DOTA [10, 11]. Especially, DOTA shows sensitivity towards harsh radiolabelling conditions, in particular, heat due to the easily oxidised Met-residue in the peptide sequence. Minigastrin analogues are also very interesting because of their targeting properties to the cholecystokinin 2 receptor, which is overexpressed in various tumour tissue-like medullary thyroid carcinoma (MTC) and gastrointestinal stromal tumours (GIST). We here report on major characteristics of an AAZTA-MG conjugate, namely [AAZTA^0^, D-Glu^1^, desGlu^2–6^]-minigastrin (AAZTA-MG), with respect to radiolabelling and in vitro and in vivo properties.

## Methods

### Reagents

For solid-phase peptide synthesis (SPPS), a Rink amide MBHA resin (100–200 mesh, Novabiochem, La Jolla, CA, USA) with a 9-Fluorenylmethoxycarbonyl (Fmoc) protective group at the *N*-terminal end was used. All amino acids (Novabiochem) were also protected by Fmoc at the amino side and the carboxyl-, amine-, hydroxyl groups, if existing, by an additional protecting group. Coupling reagents 1-Hydroxy-7-azabenzotriazole (HOAt) and O-(7-azabenzotriazol-1-yl)-1,1,3,3-tetramethyluronium hexafluorophosphate (HATU) were purchased from GenScript Corporation (Piscataway, NJ, USA). All other chemicals were obtained from VWR International GmbH (Vienna, Austria) or Sigma-Aldrich Handels GmbH (Vienna, Austria). 6-[Bis(carboxymethyl)]amino]-6-(9-carboxynonyl)-1,4-diazepane-1,4-diacetic acid, tetra-*t*-butyl ester (CN-AAZTA(tBu)_4_) was prepared according to [6].

^68^Ga-chloride was obtained from a commercial ^68^Ge/^68^Ga-generator (Modell IGG100, Eckert & Ziegler Isotope products, Berlin, Germany) with a nominal activity of 1850 MBq. For elution of the generator, 0.1 M HCl (Rotem Industries, Israel) was used. ^177^Lu-chloride in 0.04 N HCl was obtained from ITG (Munich, Germany) and ^111^In-chloride in 0.05 N HCl from Mallinckrodt Medical (Petten, NL).

### Analytical methods

Reversed-phase high-performance liquid chromatography (RP-HPLC) analysis was performed with an UltiMate 3000 RS UHPLC pump, UltiMate 3000 RS Column Compartment (column oven temperature was set at 25 °C), UltiMate 3000 Variable Wavelength Detector (Dionex, Germering, Germany) and a radio detector (GabiStar, Raytest; Straubenhardt, Germany). An ACE 3 μm C_18_ 150 × 3.0 mm (ACE, Aberdeen, Scotland) with a flow rate of 0.6 mL/min, and UV detection at 220 nm was used. The single runs were performed with the following acetonitrile (ACN)/H_2_O/0.1 % trifluoroacetic acid (TFA) gradient: 0.0–1.0 min 0 % ACN, 1.0–10.0 min 0–50 % ACN, 10.0–11.0 min 50–80 % ACN, 11.0–13.0 min 80 % ACN.

Purification was performed by preparative HPLC system (Gilson 322 Pump, Gilson UV/VIS-155) with an Eurosil Bioselect 300 × 8 mm (DF 156) and an Eurosil Bioselect 300–5 C_18_ A pre-column. A flow rate of 2 mL/min and UV detection at 220 nm with the following ACN/H_2_O/0.1 % TFA gradient was applied: 0.0–1.0 min 20 % ACN, 1.0–25.0 min 20–60 % ACN, 25.0–26.0 min 60 % CAN. The single fractions were collected by a PrepFC™ automatic fraction collector (Gilson, Middleton, WI, USA).

Thin layer chromatography (TLC) analysis was performed with silica gel impregnated glass fibre sheets (ITLC-SG stripes; Varian, Lake Forest, CA, USA) as stationary phase and sodium citrate monohydrate (0.21 g/mL, pH 5) as mobile phase and analysed using TLC scanner (Scan-RAM™, LabLogistic, Sheffield, UK).

The radioactivity of all samples was measured in a 2480 Automatic Gamma counter Wizard^2^ 3” (Perkin Elmer, Waltham, MA, USA).

### Peptide analysis by mass spectrometry

For mass spectrometry a CESI 8000 (Sciex, Brea, CA, USA) equipped with a polyethyleneimine-coated capillary (total length; 100 cm, i.d.; 30 μm, o.d.; 150 μm) was coupled via an ESI module to an LTQ Orbitrap XL ETD (Thermo Scientific, Vienna, Austria) [12]. Peptides were diluted in 0.1 % formic acid and analysed by direct infusion mass spectrometry using a flow rate of 200 nL/min. Spectra were acquired in the Orbitrap at a resolution of *R* = 60,000; the AGC target value was 1,000,000; maximum ionization time was set to 100 ms.

### Peptide synthesis and AAZTA coupling

**Fig. 1:**
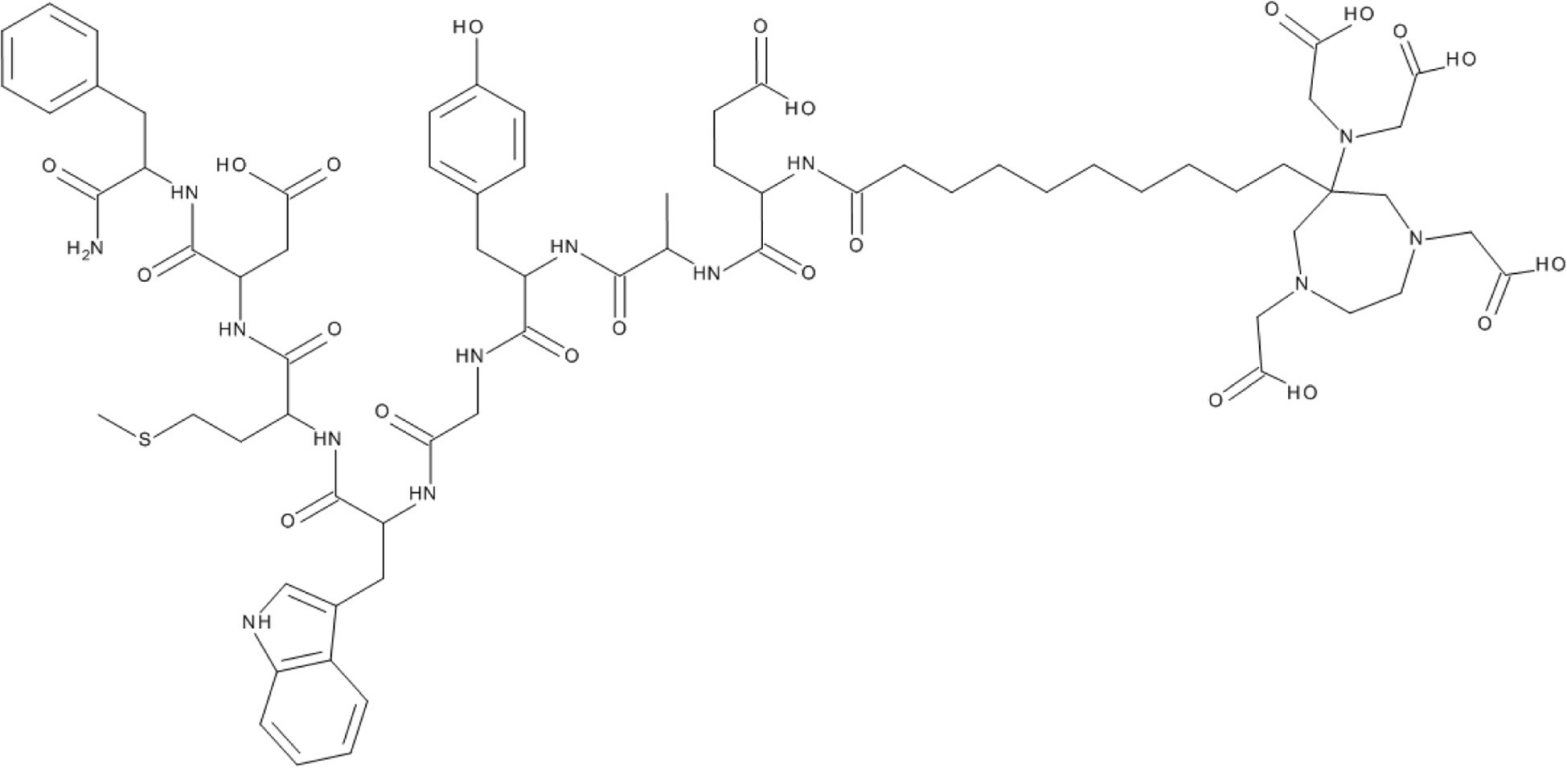
Structure of AAZTA-MG (D-Glu-Ala-Tyr-Gly-Trp-Met-Asp-Phe-NH_2_)

Synthesis of AAZTA-MG (Fig. 1) was performed using an Fmoc SPPS technique. Furthermore, 125 mg of Rink Amide resin (62.5 μmol) and 10 mL of dichloromethane (DCM) were mixed in a reaction tube. After a washing step with dimethylformamide (DMF) (3× with 10 mL for 3 min), the Fmoc protected group was cleaved by adding 10 mL of piperidine/DMF (v/v; 20/80) (2× with 10 mL for 5 min, 1× with 10 mL for 15 min) and another washing step with *N*-Methyl-2-pyrrolidone (NMP) (6× with 10 mL for 3 min).

Amino acid (fivefold excess) coupling was performed by HATU/HOAt activation in NMP at pH 8 to 9 and a reaction time of 1 to 4 h. For the amino acids Asp and Glu, an OtBu side chain protection was applied, whereas for Trp BOC and for Tyr, tBu protection groups were used.

After removing the last Fmoc protection group from the final amino acid, the AAZTA-C_9_ chelator CN-AAZTA(tBu)_4_ was coupled using a threefold excess of the chelator activated by HATU/HOAt in 10 mL NMP for 10 min at pH >10. The solution was then shaken for 18 h overnight and washed as a last step with NMP.

To cleave the peptide from the resin, a solution of TFA/Triisopropylsilane/H_2_O (v/v/v; 95/2.5/2.5) was added and left to react for 1 h, followed by ether precipitation and a drying step. The resulting peptide was dissolved in 35 % ethanol, purified by RP-HPLC and characterised by ESI-MS (see also Additional file 1).

### Radiolabelling

All radiolabelling experiments were performed at RT with reaction times of about 10 min. The radiolabelling yield was determined by radio-HPLC or radio-ITLC.

For ^68^Ga-labelling 10 μg AAZTA-MG (20 μL, 0.5 mg/mL), 30 μL sodium acetate trihydrate buffer (1.14 M, pH 4.5) and 100 μL of ^68^Ga-eluate (0.1 M HCl, 25 MBq) were used. ^177^Lu-labelling was done with 10 μg AAZTA-MG (20 μL, 0.5 mg/mL), 40 μL ascorbic acid buffer (300 μM, pH 4.5) and 40 μL of ^177^Lu (0.05 M HCl, 60 MBq). Labelling of AAZTA-MG with ^111^In-chloride was performed using 10 μg peptide (20 μL, 0.5 mg/mL), 100 μL ascorbic acid buffer (300 μM; pH 4.5), and 30 μL ^111^In (0.05 M HCl, 30 MBq). For receptor binding affinity studies (IC_50_), the -minigastrin analogue CP04 [1,4,7,10-tetraazacyclododecane-*N*,*N’*,*N”*,*N’”*-tetraacetic acid (DOTA)-DGlu-DGlu-DGlu-DGlu-DGlu-DGlu-Ala-Tyr-Gly-Trp-Met-Asp-Phe-NH_2,_ PiCHEM, Graz, Austria) was labelled with ^177^Lu as follows: 10 μg AAZTA-MG (10 μL, 1 mg/mL), 50 μL ascorbic acid buffer (300 μM; pH 4.5), and 10 μL ^177^Lu (0.05 M HCl, 160 MBq) were left to react for 10 min at RT.

### Distribution coefficient (logD)

50 μL of radiolabelled peptide solution (approx. 2 MBq; 330 pmol peptide) was added to 450 μL PBS and 500 μL octanol. The mixture was vortexed using a MS 3 basic vortexer (IKA, Staufen, Germany) for 20 min at 1400 rpm and hereafter centrifuged for 2 min at 4500 rpm. Out of both phases, 200 μL of sample were transferred into plastic vials, measured in the gamma counter and logD values were then calculated.

### Stability assay

To verify the stability of radiolabelled AAZTA-MG, tests were performed in the following four different media: ethylenediaminetetraacetic acid (EDTA), diethylenetriaminepentaacetic acid (DTPA) solutions in 1000-fold excess over the peptide, human plasma and PBS as control. The labelling solution was diluted to 1 mL and 50 μL of that solution incubated with 150 μL of the challenging solution. After 60, 120 and 240 min, 5 μL of the sample were analysed by TLC with citrate buffer (0.21 g/mL, pH = 5) as mobile phase. The strips were cut into three evenly divided pieces and measured in a gamma counter to determine the free radioligand (solvent front) to labelled peptide (origin) ratio.

### Protein binding

For this test radiolabelled peptide solution was diluted to 1 mL with PBS. From this solution, 150 μL were incubated in 850 μL fresh human plasma and in 850 μL of PBS as control. After 30, 60 and 120 min, 25 μL of the solution were analysed by size-exclusion chromatography using MicroSpin G-50 Columns (Sephadex G-50, GE Healthcare, Vienna, Austria). First, the MicroSpin columns were centrifuged at 2000 crf for 1 min to remove the storage buffer, and then, the 25 μL were added and centrifuged again for 1 min with 2000 crf. Both, column (containing the “free” radiolabelled peptide) and eluate (containing the protein-bound fraction), were measured in the gamma counter.

### Receptor binding affinity (IC_50_)

A human transfected cell line was used with two different expression properties of the cholecystokinin (CCK) receptor: CCK2+ (A431-309CCK2R; receptor-positive cells) and CCK2− (A431-609; mock transfected control cells) cells [13].

In vitro binding affinities of AAZTA-MG were determined by using CCK2+ cell membranes and ^177^Lu-labelled CP04 (a minigastrin analogue with high affinity to the CCK2 receptor) as radioligand incubated with increasing concentrations of the competitor. As competitor AAZTA-MG and [^nat^Lu]- and [^nat^Ga]-AAZTA-MG were used. The latter were prepared by incubating 10 μg AAZTA-MG with a tenfold molar excess of Lu-chloride in ascorbic acid buffer and 100 μl Ga-bromide in sodium acetate buffer for 10 min, respectively.

The 96-well plates (MulitScreen Filter Plates, HTS) were washed with 250 μL 10 mM TRIS buffered saline (pH 7.3). CCK2 cells were diluted in 20 mM HEPES buffer containing 1 M MgCl_2_—in this hypoosmotic solution the cells were cracked releasing free membranes. Then, 150 μL of cell solution (ca. 8 × 10^5^ cells/mL was added into each well and incubated with 50 μL ^177^Lu-labelled CP04 (0.97 nM) for 10 min at RT. Hereafter, 50 μL of AAZTA-MG was added at increasing concentrations (0.01–1000 nM), and the plate was incubated for 1 h at 37 °C. Unbound radioligand was removed by two washes with 250 μL TRIS buffered saline, and the remaining radioactivity on the filter was measured in the gamma counter. IC_50_ values were determined by fitting the percent inhibition data using non-linear curve fitting (Origin 6.1, Northampton, MA, USA).

### Internalisation assay

For the determination of the receptor-mediated radioligand uptake, CCK2+ and CCK2− cells were counted to a density of 8 × 10^5^ cells, seeded into 6-well plates (Greiner Bio-One) and left to grow overnight at 37 °C. On the day of the experiment to each 6-well either 150 μL of PBS/0.5 % bovine serum albumin (BSA) solution (total series) or 150 μL blocking solution (1 nM pentagastrin in PBS/0.5 % BSA solution, non-specific series) for control were added. Hereafter, 150 μL of the radiolabelled peptide was added (1.3 nM AATZA-MG) into each well and incubated for 1 h at 37 °C. To stop the internalisation process medium was removed and the supernatant collected in plastic vials. The wells were washed two times with 1 mL DMEM medium containing 1 % fetal bovine serum, and again, the supernatant was collected in plastic vials (wash fraction).

The cells were rinsed twice with 1 mL ice-cold Glycine-buffer and incubated for 5 min at RT to remove membrane-bound radiolabelled peptide (membrane-bound fraction). Subsequently, the cells were lysed by addition of two times 1 mL of 2 M NaOH (internalised fraction). All collected solutions were measured in the gamma counter.

### Biodistribution

All conducted animal experiments were in compliance with the Austrian and Czech animal protection laws and with approval of the Austrian Ministry of Science (BMWFW-66.011/0049-WF/II/3b/2014) and the Czech Ministry of Education Youth and Sports (MSMT-22421/2013-12).

For normal biodistribution experiments 100 μL of [^68^Ga]AAZTA-MG and [^177^Lu]AAZTA-MG (~0.3 nmol, ~0.4 μg peptide; 1 MBq/mouse) were simultaneously injected into the tail vein of 8-to-10-week-old female BALB/c mice (*n* = 4, Charles River laboratories, Sulzfeld, Germany). The animals were sacrificed by cervical dislocation 60 min post injection (p.i.). Organs (heart, stomach spleen, liver, pancreas, kidneys and intestine), blood and muscle tissue were removed and weighted. Activity of the samples was measured simultaneously in the gamma counter (^177^Lu as well as ^68^Ga) using the respective gamma-energy windows, and the results expressed as percentage of injected dose per gram tissue (% ID/g).

For biodistribution studies in tumour xenograft-bearing mice, female athymic BALB/c nude mice (Charles River Laboratories, Sulzfeld, Germany) were used (*n* = 4 per group). For the induction of tumour xenografts, mice were injected subcutaneously near the front shoulder with 2 × 10^6^ cells (CCK2+) and the same amount of CCK2− cells as a control subcutaneously in 100 μL DMEM + 100 μL matrigel. The tumours were allowed to grow until they had reached a volume of 0.3 to 0.6 cm^3^. 1 MBq (~0.3 nmol, ~0.4 μg peptide) of either ^68^Ga or ^111^In-labelled AAZTA-MG (*n* = 4) was administered retroorbitally. After 60 min, mice were sacrificed, and accumulated radioactivity was determined in organ and tumour tissues and the results expressed as percentage of injected dose per gram tissue (% ID/g).

### μPET/CT

The positron emission tomography (PET) and computed tomography (CT) images were acquired with an Albira PET/SPECT/CT small animal imaging system (Bruker Biospin Corporation, Woodbridge, CT, USA). Radiolabelled tracer ([^68^Ga]AAZTA-MG ~ 5 MBq) was administered retroorbitally into tumour xenograft-bearing nude mice with a positive tumour at the left flank (2*10^6^ CCK2+ cells) and a negative tumour at the right flank (2*10^6^ CCK2− cells). Mice were subsequently anaesthetized with isoflurane (FORANE, Abbott Laboratories, Abbott Park, IL, USA; 2 % flow rate) and were kept under anaesthesia during the imaging process. Static PET/CT imaging was carried out at 30, 45 and 60 min p.i. A 10-min PET scan (axial FOV 148 mm) was performed, followed by a CT scan (axial FOV 65 mm, 45 kVp, 400 μA, at 600 projections). Dynamic imaging was carried out immediately after the injection of ^68^Ga labelled tracer for 90 min (5-min PET scan per frame). Scans were reconstructed with the Albira software (Bruker Biospin Corporation, Woodbridge, CT, USA) using maximum likelihood expectation maximization (MLEM) and filtered backprojection (FBP) algorithms [14, 15]. After reconstruction, the acquired data were viewed and analysed with PMOD software (PMOD Technologies Ltd., Zurich, Switzerland). The 3D images were obtained using VolView software (Kitware, Clifton Park, NY, USA).

## Results

### Peptide synthesis

Solid-phase peptide synthesis of AAZTA-MG resulted in 1.5 mg of the conjugate which correlates to a theoretical yield of 8.4 %. The purified peptide contained 4 % of oxidised methionine which could not be removed by further purification. ESI-MS [M + H] = 1516.67 [C_71_H_97_N_13_O_22_S; exact mass: 1515.66 (calculated)] and HPLC traces of synthesis products and the purified peptides are provided in the Additional file 2, proving identity and purity of the AAZTA-MG bioconjugate.

### Radiolabelling

The AAZTA chelator showed excellent labelling performance resulting in specific activities for ^68^Ga (15.1 GBq/μmol), ^177^Lu (27.2 GBq/μmol) and ^111^In (4.5 GBq/μmol), respectively, at pH of 4.0 to 4.5. With all radionuclides, high radiochemical yields typically of about 95 % could be achieved. Higher reaction temperatures (80 to 90 °C) showed no significant differences to labelling at RT.

### In vitro characterisation

Distribution coefficient (logD) for [^68^Ga]AAZTA-MG was −3.6 (±0.18 SD; *n* = 6) and for [^177^Lu]AAZTA-MG −3.73 (±0.17 SD; *n* = 2), respectively. The difference between these two was negligible, which indicates that the peptide labelled with both radiometals is a highly hydrophilic compound. The chelator AAZTA did not dramatically reduce hydrophilicity. Stability tests of [^68^Ga]AAZTA-MG (*n* = 3) showed an instability in human plasma and a slight transchelation with EDTA and DTPA. This indicates some release of ^68^Ga, which also was observed in the biodistribution study, where blood levels with the ^68^Ga-compound were increased. In comparison, [^177^Lu]AAZTA-MG (*n* = 3) showed a high stability in PBS, EDTA and human plasma but some transchelation with DTPA (Table 1).

**Table 1:** Protein binding (*n* = 6) and stability (*n* = 3) values after 30, 60 and 120 min incubation at RT for ^68^Ga- and ^177^Lu-labelled compounds

Incubation time [min]	A [%]		B [%]		C [%]		D [%]	
	^68^Ga	^177^Lu	^68^Ga	^177^Lu	^68^Ga	^177^Lu	^68^Ga	^177^Lu
30	9.5 ± 0.4	4.9 ± 0.7	96 ± 2.1	99 ± 0.27	94 ± 1.6	92 ± 0.24	90 ± 3.2	97 ± 0.47
60	13.0 ± 1.2	4.9 ± 1.0	95 ± 2.7	98 ± 0.24	91 ± 1.6	87 ± 0.31	88 ± 2.1	98 ± 0.12
120	15.8 ± 1.8	3.7 ± 0.8	92 ± 5.5	98 ± 0.12	90 ± 0.5	77 ± 0.72	81 ± 7.9	97 ± 2.12

Data are shown as means ± SD

*A* serum protein binding; *B* stability in EDTA solution; *C* stability in DTPA solution; *D* stability in fresh human serum

Protein binding in human plasma was significantly higher (*p* = 0.01 *t* test) for [^68^Ga]AAZTA-MG than for [^177^Lu]AAZTA-MG. The protein-bound fraction increased over time for the ^68^Ga-labelled compound from 9.5 to 15.8 %, indicating a slow release of ^68^Ga. In contrast to that, the ^177^Lu-labelled compound showed 4.5 % protein binding with little variation over time.

**Fig. 2:**
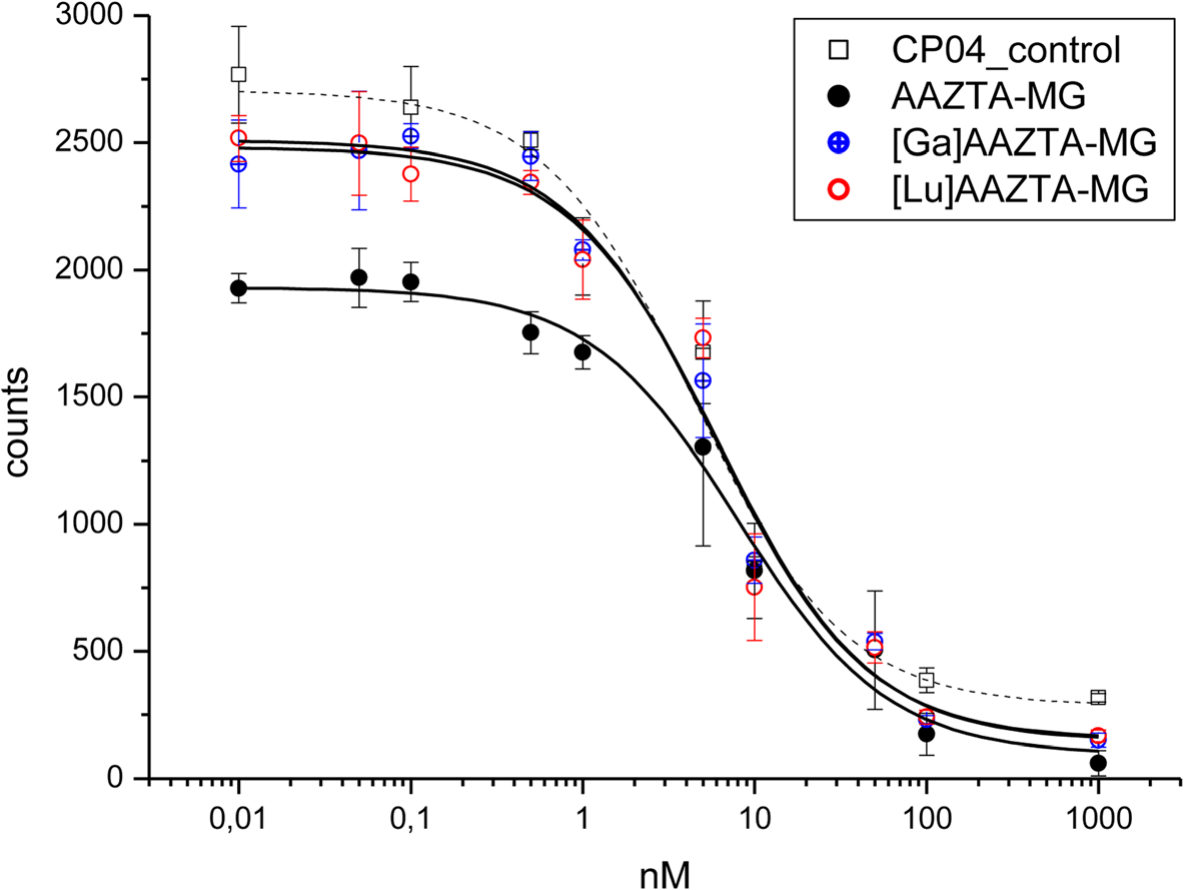
Displacement curves of [^177^Lu]CP04 binding to CCK2+ cell membranes using increasing concentrations of AAZTA-MG, [^nat^Ga]AAZTA-MG, [^nat^Lu]AAZTA-MG and CP04 as control. Data are given as means ± SD

For AAZTA-MG an IC_50_ value of 10.0 nM (±3.02 SD; *n* = 6) was determined. Between [^nat^Lu] (*n* = 2; 9.44 ± 4.57 SD) and [^nat^Ga]-AAZTA-MG (*n* = 2; 8.18 ± 3.16) no significant (*p* = 0.33 *t* test) difference could be determined (Fig. 2). This indicates a high affinity of the AAZTA-MG conjugate to the CCK2 receptor independently of the introduced radiometal.

**Fig. 3:**
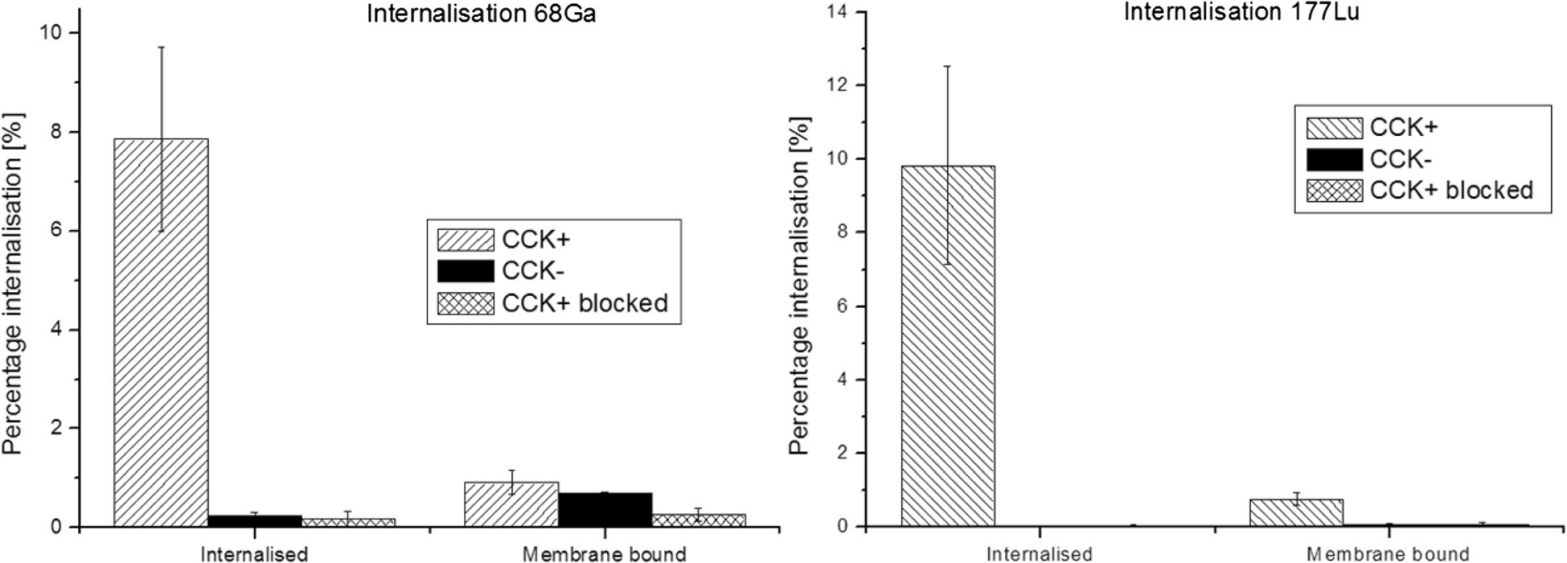
Internalisation assay of [^68^Ga]AAZTA-MG and [^177^Lu]AAZTA-MG into CCK2+, CCK2− cells and CCK+ but blocked with Pentagastrin; “Internalised” equates to internalisation ability of AAZTA-MG into the cells, and “Membrane bound” shows the adsorption to the external membrane (membrane-bound fraction); Data are shown as means ± SD

The internalisation assay of AAZTA-MG showed a specific internalisation for both radiolabelled peptides, but no relevant differences between [^68^Ga]AAZTA-MG (7.70 ± 1.85 SD; *n* = 4) and [^177^Lu]AAZTA-MG (9.79 ± 2.68 SD; *n* = 5); in both cases surface bound activity was <10 % of internalised activity. Blocking receptors with pentagastrin reduced internalised activity below 1 % (non-specific binding) (Fig. 3).

### In vivo characterisation

#### Biodistribution of labelled AAZTA-MG in normal mouse and in a tumour xenograft mouse model

Biodistribution in BALB/c mice was determined 60 min p.i. The radioactivity in different tissues of normal mice (^111^In- vs. ^68^Ga-AAZTA-MG) and tumour xenografted mice ([^177^Lu]- vs. [^68^Ga]-AAZTA-MG) are shown in Table 2. Slight differences between the ^68^Ga-, ^177^Lu- and ^111^In-labelled peptide conjugates were observed: [^177^Lu]AAZTA-MG and [^111^In]AAZTA-MG showed high radioactivity accumulation in intestine and liver, which indicates hepatobiliary excretion combined with low blood levels. In contrast, [^68^Ga]AAZTA-MG showed high blood levels correlating with the limited stability in vitro and high protein binding of the ^68^Ga-compound. All three different labelled compounds showed very low accumulation in kidneys, comparable to other studies with MG11 [16].

**Table 2:** Biodistribution data of AAZTA-MG labelled with different radiometals 1 h p.i. in different mouse models

	^111^In-AAZTA-MG	^68^Ga-AAZTA-MG	^177^Lu-AAZTA-MG	^68^Ga-AAZTA-MG
	CCK+/− BALB/c nude mice	CCK+/− BALB/c nude mice	BALB/c nude mice	BALB/c nude mice
Blood	0.14 ± 0.03	0.38 ± 0.07	0.10 ± 0.09	2.90 ± 1.39
Spleen	0.14 ± 0.02	0.13 ± 0.01	0.11 ± 0.04	0.91 ± 0.34
Pancreas	0.13 ± 0.03	0.14 ± 0.02	0.12 ± 0.04	0.68 ± 0.28
Stomach	0.61 ± 0.11	0.47 ± 0.03	1.18 ± 0.13	0.95 ± 0.22
Intestine	8.62 ± 1.24	0.61 ± 0.08	19.14 ± 3.40	2.43 ± 0.32
Kidneys	1.79 ± 0.32	2.51 ± 0.66	1.39 ± 0.73	3.37 ± 0.43
Liver	2.46 ± 0.96	0.42 ± 0.07	3.05 ± 1.31	1.48 ± 0.26
Heart	0.12 ± 0.09	0.15 ± 0.04	0.07 ± 0.06	0.88 ± 0.32
Lung	0.21 ± 0.06	0.33 ± 0.07	0.14 ± 0.05	1.59 ± 0.47
Muscle	0.15 ± 0.18	0.07 ± 0.03	0.10 ± 0.17	0.36 ± 0.23
Femur	0.20 ± 0.11	0.11 ± 0.02	0.21 ± 0.18	0.74 ± 0.42
CCK2+ tumour (left flank)	1.74 ± 0.80	1.51 ± 0.53		
CCK2− tumour (right flank)	0.33 ± 0.11	0.44 ± 0.06		

Data are shown as %ID/g (mean ± SD, *n* = 4)

Biodistribution of [^68^Ga]AATZA-MG and [^111^In]AATZA-MG in the tumour xenograft model revealed a comparable uptake in the CCK2+ tumour (^68^Ga = 1.5 %ID/g; ^111^In = 1.74 %ID/g), which is about fourfold higher than in the CCK2− tumour (^68^Ga = 0.44 %ID/g; ^111^In = 0.33 %ID/g). This indicates a specific uptake in the receptor-positive tumour tissue.

### μPET-CT imaging

**Fig. 4:**
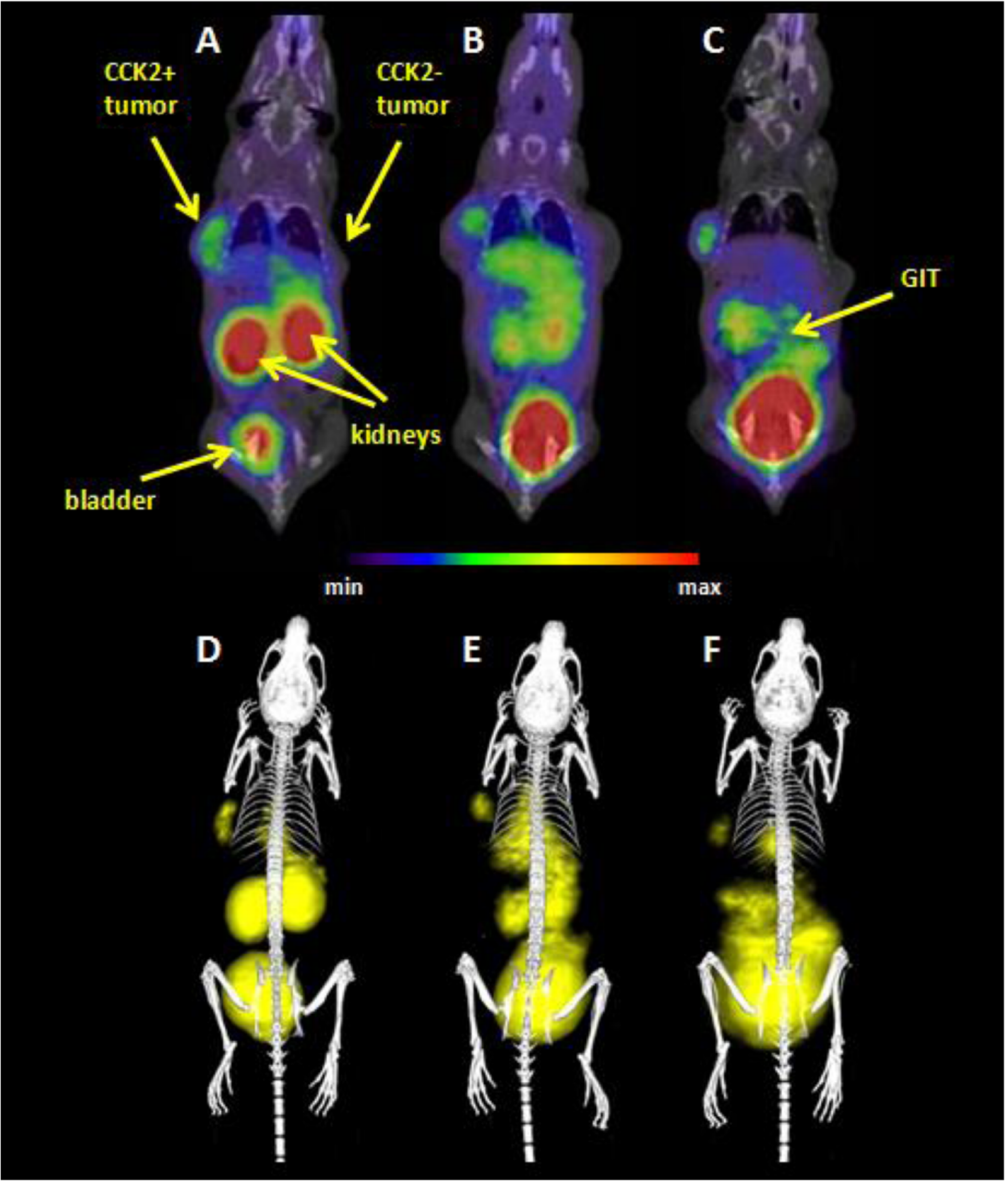
Static μPET/CT images of [^68^Ga]-AAZTA-MG in CCK+/− tumour xenograft-bearing BALB/c nude mice 30 (**a**, **d**), 45 (**b**, **e**) and 60 (**c**, **f**) min p.i.; coronal slices (**a**, **b**, **c**) and 3D volume rendered images (**d**, **e**, **f**). (supine position; injected dose ~5 MBq; anaesthesia: 2 % isoflurane; scan duration: 10-min PET scan followed by 30-min CT scan)

The μPET scans with [^68^Ga]AAZTA-MG showed good targeting properties for CCK2− receptor-expressing tissue corresponding with the data from biodistribution studies. Radioactivity in kidneys and bladder could be explained by the main renal excretion way. Also, specific uptake in the CCK2+ tumours was observed with no uptake in the CCK2− tumours. Images obtained at three different time points (30, 45, and 60 min p.i.) are shown in Fig. 4. The not only the kidneys and bladder shown were clearly visualized but also the CCK2+ tumour. In contrast, the CCK2− control tumour did not show any signal above background. Over time, washout from kidneys and some hepatobiliary excretion which was predicted from the biodistribution studies can be seen in the images.

**Fig. 5:**
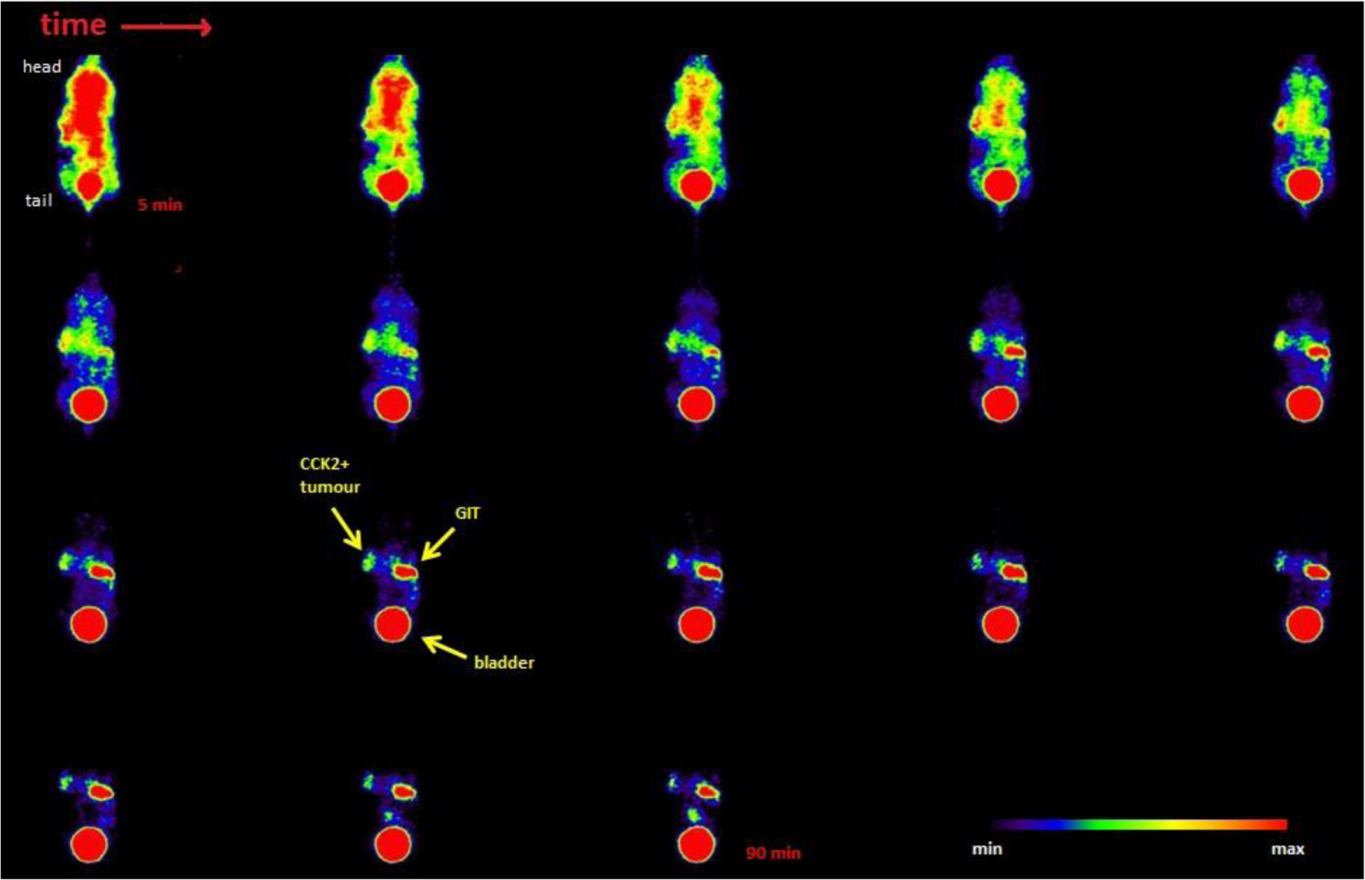
90 min dynamic PET scan; consecutive 5 min coronal sections show accumulation in the CCK2+ tumour (*left flank*), bladder and gastrointestinal tract. Kidneys are located outside the section plane

A dynamic scan over 90 min but only in one slice of PET scan without CT is shown in Fig. 5. High radioactivity accumulation in the gall bladder and the tumour was observed. Kidneys are not shown in this slice, but a distribution in the gastrointestinal tract was seen over time, which also confirms the biodistribution results.

## Discussion

Choosing appropriate chelators gives radiopharmaceutical science the opportunity to utilize different radionuclides for diagnosis and to move established radiopharmaceuticals towards therapeutic applications, enabling a theranostic approach. Particularly, the chelator AAZTA shows a novel versatile way of binding trivalent radionuclides, providing mild radiolabelling conditions combined with high-specific activities. So far only few data are available on the properties of AAZTA conjugated biomolecules or peptides.

We chose the minigastrin analogue MG11 as a model peptide, since gastrin is an interesting peptide for both diagnostic and therapeutic applications in rare malignant diseases (MTC, GIST) [17–19], and this particular peptide was already used in combination with a number of alternative chelating systems. Minigastrin represents a peptide which is sensitive to extensive heating steps, as methionine in its peptide sequence is prone to oxidation leading to loss of receptor binding. In case of the chelator AAZTA, no heating for radiolabelling is required, and the problem of Met-oxidation can be almost eliminated. In our study, all radiolabelling experiments could be carried out at RT whereas for examples for DOTA radiolabelling temperatures of more than 80 °C are usually needed.

Coupling of the AAZTA derivative to MG11 was successful and proven by ESI-MS analysis. In this preliminary study, no optimization of the synthesis was performed, which is why relatively low yields were achieved; however, these low yields were sufficient for the initial characterisation of the AAZTA-MG bioconjugate.

Radiolabelling results were overall very good and showed quantitative radiolabelling at high-specific activities for ^177^Lu, ^68^Ga and ^111^In, respectively.

The logD values for [^68^Ga]AAZTA-MG and [^177^Lu]-AAZTA-MG indicate a highly hydrophilic character of the complexes. This high hydrophilicity would typically result in renal excretion of these peptides. Biodistribution and μPET-CT studies, however, revealed partly excretion via the hepatobiliary route. This may be a consequence of the rather lipophilic linker (aliphatic C9 chain) used in the applied AAZTA derivatives or potentially a result of hepatobiliary excretion of certain metabolites, considering the limited metabolic stability of MG11-based peptide conjugates. Overall high stability in different solutions was found for both radiometal labels, with some decomposition in human plasma for [^68^Ga]AAZTA-MG. This corresponds to the high blood levels seen in the biodistribution study resulting in lower tumour-to-background ratios in the μPET scans. This was also confirmed by the higher protein-binding values of [^68^Ga]AAZTA-MG as compared to its ^177^Lu counterpart. The amount of ^68^Ga bound to serum proteins was increasing over time which can be explained by transchelation to transferrin due to a low complex stability. This is in contrast to the findings of Baranjay et al. [20] who reported excellent stability of AAZTA complexes with stability constants (logK) of 22.18 for Ga^3+^ and 29.58 for In^3+^, which compares well with the stability constant of ^68^Ga-DOTA with 21.3 and 23.9, respectively. Waldron et al. [8] also found excellent stability of [^68^Ga]-AAZTA complexes labelled at pH 4 and above. However, they also describe unstable “kinetically trapped” side products with low stability towards challenging solutions when labelled at pH 3.3 or lower. We were not able to detect such side products by HPLC with our [^68^Ga]-AAZTA-MG, radiolabelled at pH 4.5, but cannot fully exclude their presence. Nevertheless, in a recent comparison of different ^68^Ga-chelators, AAZTA also showed some trend to release the radiometal in transchelation experiments [21]. Despite the high thermodynamic stability of AAZTA-metal chelates, in comparison to DOTA, our studies indicate limitations in kinetic stability in particular of [Ga]AAZTA complexes.

Therefore, dedicated applications with ^68^Ga chelators like DOTA or NOTA may be preferable [22, 23]. In contrast to these findings for [^177^Lu]AAZTA-MG, only limited transchelation towards DTPA was observed, but no indication for in vivo instability was found; also, protein binding was neglectable.

The low IC_50_ values of AAZTA-MG showed high affinity for the CCK2 receptor, comparable to other results of MG11 conjugated to DOTA [24]. Also, binding studies with the corresponding ^nat^Ga- and ^nat^Lu-labelled compounds revealed no difference to the “metal-free” peptide. Specific cell uptake after 60-min incubation resulted in high-specific internalisation for both ^68^Ga- and ^177^Lu-labelled AAZTA-MG conjugates with no significant difference between the two radiometals.

Biodistribution in normal BALB/c mice for ^177^Lu-labelled AAZTA-MG showed comparable low retention in the kidneys but higher hepatobiliary excretion to DOTA-MG11 [16]. Biodistribution of ^68^Ga- and ^111^In-labelled peptide was performed in BALB/c mice and CCK2 tumour xenografted nude mice including μPET studies. They revealed a significant (^68^Ga *p* = 0.007; ^111^In *p* = 0.01 *t* test) difference in accumulation in CCK2+ vs. CCK2− tumours (Table 2). Accumulation of peptide in the CCK2+ tumour was well above background and allowed to visualize the size of the tumour whereas almost no radioactivity was detected in the CCK2− control tumour. These findings are overall comparable to other μPET scans with DOTA-MG analogues with the exception of a higher uptake in the intestinal tract [22]. However, tumour uptake with 1.74 % ID/g was somewhat lower as for [^111^In]DOTA-MG11 with 3.04 % ID/g in the same tumour model [16]. This cannot be explained by receptor binding or internalisation behaviour which was comparable for both compounds, therefore probably related to the differences in pharmacokinetics by introducing another bifunctional chelator.

The tumour-to-kidney ratio of 0.9 for ^111^In and 0.6 for ^68^Ga is also comparable to DOTA conjugated to MG11 [25]. For [^68^Ga]AAZTA-MG, higher blood levels were observed. This indicates an in vivo instability of the [^68^Ga]AAZTA complex and correlates with our in vitro tests, in particular, the high protein binding and instability in human blood serum. It also resulted in a tumour-to-blood ratio of about 4 with 1.5 % ID/g in CCK2+ tumours as compared to a blood level of 0.4 % ID/g. This is in contrast to Tornesello et al. [25] who reported higher contrast for the [^68^Ga]DOTA-MG11 over the [^111^In]DOTA-MG11. For [^111^In]AAZTA-MG, the tumour-to-blood ratio was considerably higher with a value of 12, which seems slightly lower compared to [^111^In]DOTA-MG11 in the same tumour model [16]. Again [^111^In]AAZTA-MG also showed considerable higher excretion via the intestinal tract, which could be explained by the aliphatic linker used for AAZTA conjugation. This could be addressed by modifying the structure of the AAZTA conjugate, e.g., by truncating the spacer length between peptide and chelator to eliminate intestinal activity. Also, conjugation to other minigastrin peptide structures could improve tumour targeting and pharmacokinetic properties, e.g. Laverman et al. [16] showed that sic D-Glu in the amino acid sequence led to excellent tumour targeting combined with increased metabolic stability and improved pharmacokinetics including low kidney retention.

## Conclusions

In this study, we were able to couple the novel mesocyclic chelator AAZTA to a minigastrin structure (MG11) demonstrating its use for radiopharmaceutical applications. It was shown that radiolabelling with different radiometals, including ^68^Ga, ^177^Lu and ^111^In, is possible with a high yield at mild conditions and high-specific activities comparable to the currently most widely used chelator DOTA. Some limitations were observed that require further research efforts. Even though [^68^Ga]AAZTA-MG showed good tumour targeting, the observed instability in blood could be challenging for diagnostic imaging. Also, the partial excretion of [^177^Lu]- and [^111^In]-AAZTA-MG through the hepatobiliary route is certainly suboptimal for a theranostic approach. Nevertheless AAZTA provides a new opportunity for the development of radiopeptides and other targeted radiopharmaceuticals, in particular for use as a theranostic agent.

## Additional files

Additional file 1: Figure S1. (molecular weight = 1,515 g/mol). Additional file 2: Figure S2. HPLC chromatograms at different purification steps. Top: Peptide after cleavage of the rink amid resin; Middle: Fraction of the purification from the preparative HPLC; Bottom: pure peptide with <4 % oxidised content.
