# Supplementary figures and images for: Influence of a novel, versatile bifunctional chelator on theranostic properties of a minigastrin analogue
Source: EJNMMI Res. 2015 Dec 15;5:74. doi: 10.1186/s13550-015-0154-7 (PMC4679714; doi:10.1186/s13550-015-0154-7)

Additional file 1: Figure S1: ESI-MS spectrum of AAZTA-MG


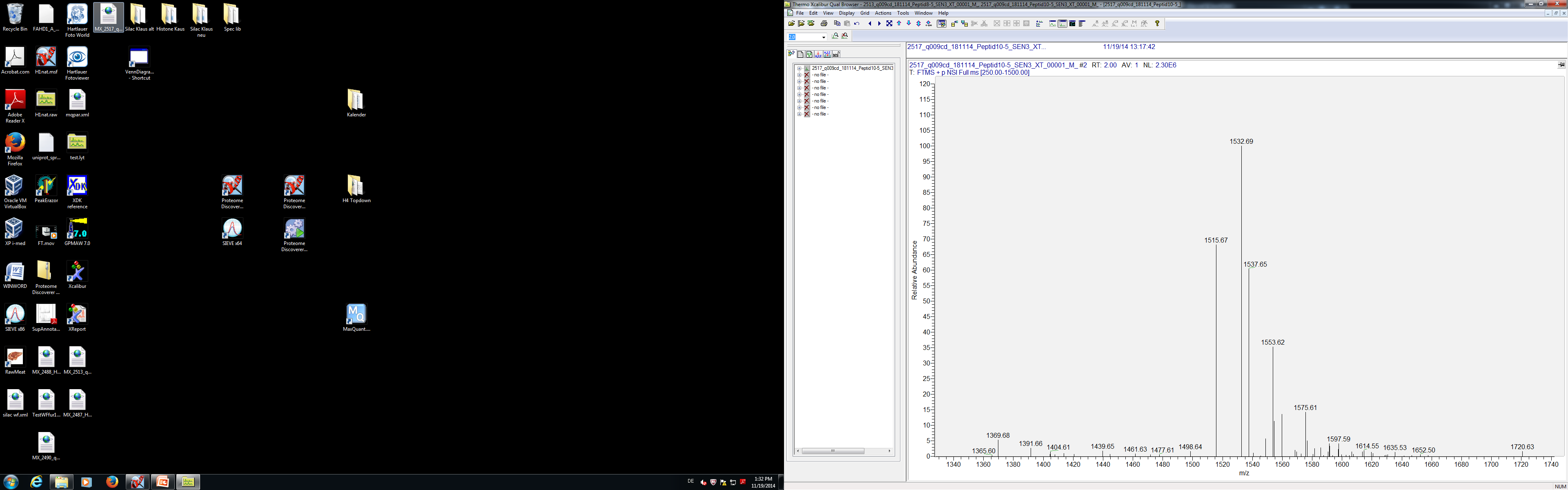

Supplement: Additional file 1: Figure S1. — (molecular weight = 1,515 g/mol). [file 13550_2015_154_MOESM1_ESM.docx]

Additional file 2: Figure S2: HPLC chromatograms at different purification steps


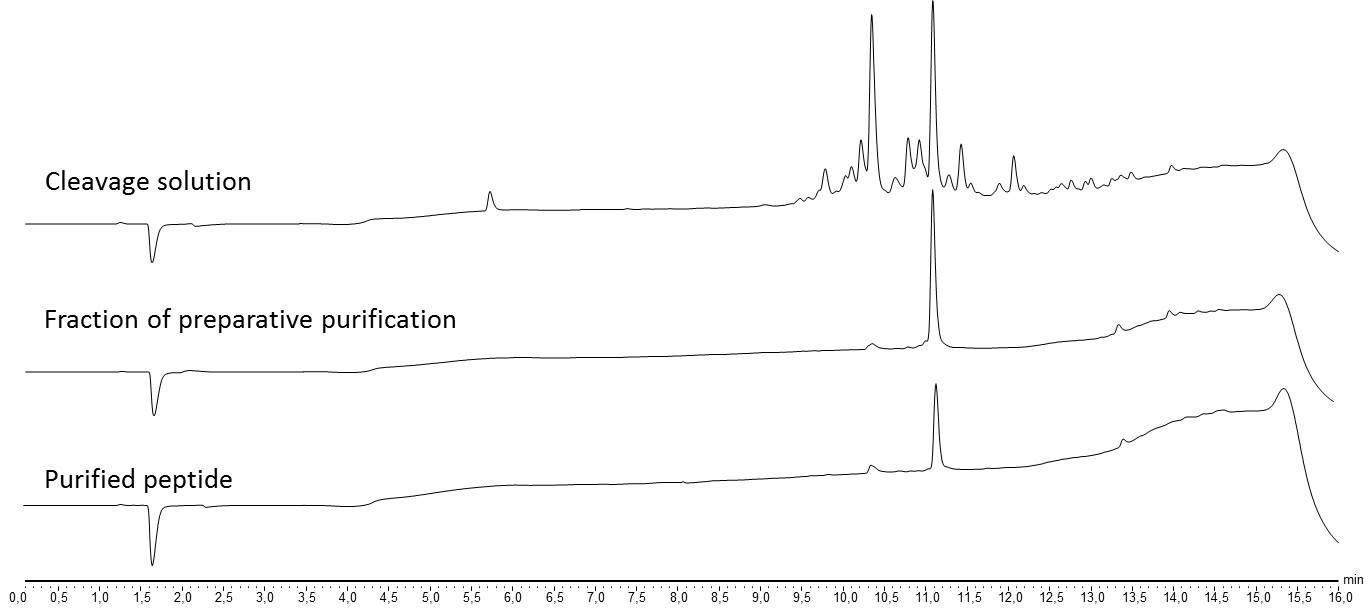

Supplement: Additional file 2: Figure S2. — HPLC chromatograms at different purification steps. Top: Peptide after cleavage of the rink amid resin; Middle: Fraction of the purification from the preparative HPLC; Bottom: pure peptide with <4 % oxidised content. [file 13550_2015_154_MOESM2_ESM.docx]
